# Efficacy of Single Stretching Session of Iliopsoas Using Proprioceptive Neuromuscular Facilitation Versus Muscle Energy Technique on Low Back Pain in Patients With Lumbar Hyper-Lordosis

**DOI:** 10.7759/cureus.27916

**Published:** 2022-08-12

**Authors:** Tasneem M Lakkadsha, Moh'd Irshad Qureshi, Rakesh K Kovela, Sakina S Saifee, Shivani S Lalwani

**Affiliations:** 1 Department of Physiotherapy, Ravi Nair Physiotherapy College, Datta Meghe Institute of Medical Science, Wardha, IND; 2 Physiotherapy, Nitte Institute of Physiotherapy, NITTE (Deemed to be University), Mangalore, IND

**Keywords:** muscle energy technique, proprioceptive neuromuscular facilitation, anterior pelvic tilting, lumbar hyper-lordosis, low back pain

## Abstract

Background and objectives

One of the most frequent conditions for which people seek physiotherapy treatment is low back pain (LBP). When the aetiology of low back pain is whittled down to mechanical factors, pelvic tilting becomes apparent. The iliopsoas muscle is the key to relieving LBP in such circumstances, and since it is tightened, we concentrated on stretching it adequately in this study. Proprioceptive neuromuscular facilitation (PNF) and muscle energy technique (MET) are two stretching techniques that we have compared for this purpose. There are many other stretching techniques available, but the evidence has proven these two to be the most effective.

Methods

The participants in the study were those between the ages of 18 and 60 who had exaggerated lumbar lordosis, or LBP, and met the inclusion criteria. There were two groups created: A and B. PNF and traditional physiotherapy was used to manage the participants in group A, and MET and traditional physiotherapy were used to manage the participants in group B. Each group underwent the same pre-and post-tests, which included the Numerical Pain Rating Scale (NPRS) to assess pain intensity; a universal goniometer to measure hip joint extension range of motion (ROM) to assess iliopsoas flexibility; and a side-lying X-ray to measure the lumbosacral angle (LSA) to determine the angle of lumbar lordosis.

Result

In both the stretching interventions, i.e., PNF and MET, there were statistically significant differences in pain, hip extension range of motion, and lumbar lordosis angle (P > 0.0001). However, for the PNF group, the difference between the pre-and post-test was greater than that for the MET group.

Conclusion

The current study, which included 100 participants, demonstrated that both PNF and MET are remarkably effective for loosening the tight iliopsoas. A comparison of both techniques showed that the PNF group had benefited significantly more than the MET group.

## Introduction

Low back pain (LBP) is frequently experienced by people who are in an activity flag, interfering with their daily jobs and activities [1-3]. There are several causes of LBP that can be specific or non-specific; however, this study focuses on one of the specific causes, i.e., the link between LBP and iliopsoas tightness, as well as the most effective stretching technique for reducing the pain severity within a single session.

The iliopsoas muscle is the most powerful hip joint flexor. It is known to link the spine to the lower extremities and has been directly linked to back pain [4]. The iliacus and psoas major make up this deep muscle [5]. This muscle often becomes tight as a result of an inactive lifestyle and leads to a reduction in the range of motion (ROM) and mobility in the joints, which can interfere with daily activities. Because these muscles are not typically stretched during daily activities, people whose jobs require them to sit for prolonged periods of time throughout the day, such as computer professionals or desk workers, are more likely to experience adaptive changes that shorten these muscles [4,6-8]. Once the iliopsoas muscle's length shortens, the spine hyper-lordoses and the pelvis tilts anteriorly, placing stress on the erector spinae and all other spinal muscles. Anterior pelvic tilting can be brought on by a number of other factors too, but is most frequently brought on by an abundance of muscles pulling on the lumbar and/or pelvic regions, causing both innominate to rotate anteriorly. This form of postural distortion pattern that affects the pelvic and low back muscles is known as the lower cross syndrome [9]. The reciprocal inhibition of the gluteus maximus brought on by iliopsoas tightness is another way to describe it [10]. The rectus femoris and quadratus lumborum, in addition to the iliopsoas and erector spinae, are crucial postural muscles that appear hypertonic in the pelvic and lower back regions. These muscles can result in lumbar lordosis and anterior pelvic tilt when they are overly tight. The pelvic and abdominal phasic muscles, which include the gluteus medius, gluteus maximus, and rectus abdominis, are in opposition to this group. It is possible that inactivity will cause phasic muscles to deteriorate. Living a sedentary lifestyle promotes the overuse of postural muscles at the expense of phasic muscles [9].

When compared to stable individuals, people with iliopsoas tightness exhibit significantly lower iliopsoas strength, constrained hip extension ROM, increased pelvic tilting, and lumbar lordosis [11]. When this muscle was stretched while supine with the knees in a semi-flexed position, there was an increase in muscle flexibility as well as a tendency for the pelvis to retrovert and neutralise [12].

There are several methods for releasing iliopsoas tension, including proprioceptive neuromuscular facilitation (PNF) [13], muscle energy method (MET) [14,15], stretching (ballistic, static), soft tissue mobilization, yoga (asanas such as Virabhadrasana, Sarvangasana, Navasana), and myofascial release.

## Materials and methods

**Figure 1:**
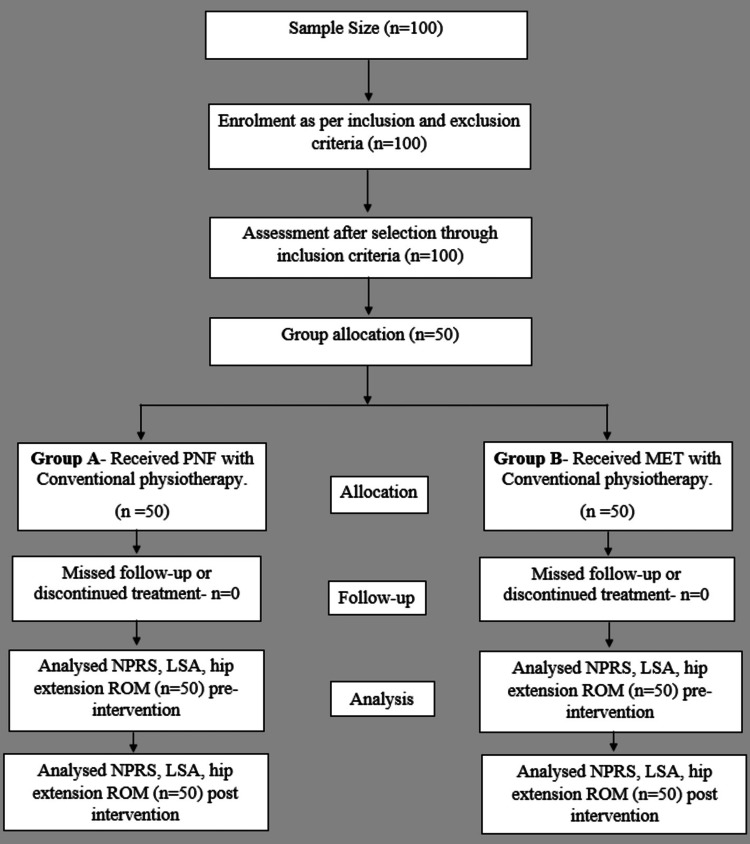
Flowchart of the study procedure

After receiving approval from the Institutional Ethics Committee of Datta Meghe Institute of Medical Sciences (DMIMS) (Deemed to be University [DU]) (Ethical permission number: DMIMS[DU]/IEC/2021/379), the study was conducted in the neuro-physiotherapy outpatient department (OPD), and the participants were drawn from the orthopedic and neuro-physiotherapy OPDs of Acharya Vinoba Bhave Rural Hospital Sawangi (Meghe), Wardha, Maharashtra. Informed consent was obtained, arbitrary data was gathered, and a preliminary evaluation was completed to determine whether or not the individuals met the inclusion and exclusion criteria. The inclusion criteria were that the participants had to be of either gender and between the ages of 20 and 65, have LBP (Numerical Pain Rating Scale [NPRS] >4), have hyper-lordosis of the lumbar spine (Lumbo-Sacral angle [LSA] >40°), be able to understand and follow instructions, be willing to participate in the study, and be able to complete the outcome measures. People with LBP (Numerical Pain Rating Scale 4), hyper-lordosis of the lumbar spine (Lumbo-Sacral angle 40°), complaints of radiculopathies with or without neurological deficits, pregnant women, people who have had surgery on their lumbar or thoracic spine, people who have anatomical deformities of the spine or chest wall, and those who are currently enrolled in another research trial for a similar illness and have non-specific LBP were all excluded from the study. The objectives and methods of the study were then explained to the qualified participants. Using the SNOSE method, the participants were randomly assigned to either group A or group B by simple random sampling. The primary researcher, a physiotherapy department intern, conducted the randomization and allocation. The study's enrolment, intervention, and assessment schedule followed the guidelines in the standard protocol items: a suggestion for conducting intervention trials [16]. The flowchart of the study procedure is depicted in Figure 1.

Outcome measures

A physiotherapy undergraduate student who was aware of the study but blinded to the intervention evaluated the following outcome measures before and after the intervention.

Numerical Pain Rating Scale

On a piece of paper, 0-10 markings were made, in which 0 was no pain and 10 was maximum pain. The NPRS assisted in quantifying the patient's low back pain intensity before and after the intervention, as well as any improvement or exacerbation. A 2-point change on the NPRS indicates a clinically significant change that exceeds the measurement error boundaries [17].

Lumbosacral Angle

In order to analyze the difference in the lumbar lordosis, we measured the LSA through an X-ray while the patient was supine (by an angle created by a line passing through the superior aspect of the S1 vertebra and a horizontal line [18]) before and after the intervention. It has been described as the gold standard in some studies [19]. According to earlier research, the normal lordosis angle is 30°, and hyper-lordosis angles are greater than 40° [20]. Lumbar lordosis angle and lumbosacral angle have been used interchangeably in the text below.

Goniometry

A goniometer is used to measure a joint's range of motion in degrees; in this case, it was used to compare the degree of hip extension before and after the intervention. It has been determined that conventional manual goniometers are reliable for use in clinics for longitudinal examinations [21].

Intervention

Measurements were made before and after the intervention. PNF and traditional physiotherapy were given to the subjects in group A, while MET and traditional physiotherapy were given to the subjects in group B. Each technique was applied three times, and both groups received traditional physiotherapy in the form of a hot pack applied to the low back region prior to treatment for ten minutes.

**Figure 2:**
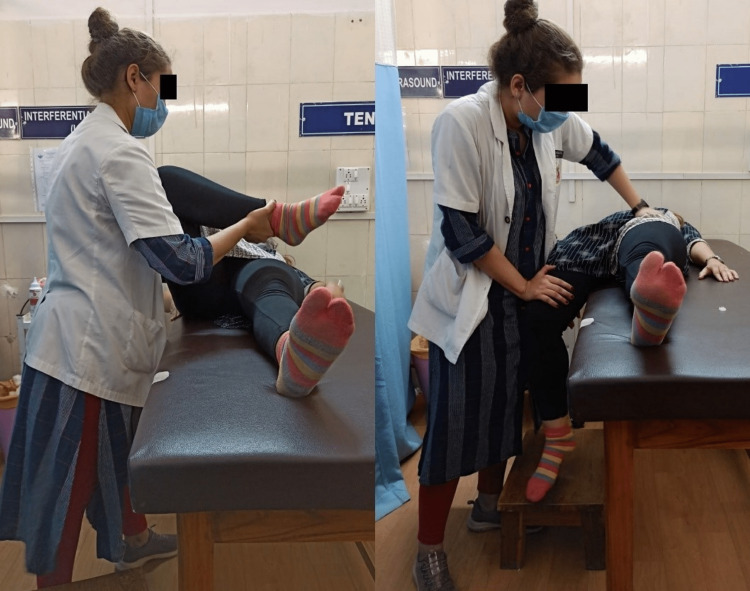
Start (left) and end (right) position for PNF stretching technique PNF: proprioceptive neuromuscular facilitation

In group A, participants received basic conventional physiotherapy along with bilateral iliopsoas stretching using the PNF hold-relax D1 movement pattern three times in a single session with a break of 2 minutes in between each repetition. The hold-relax technique was used to perform the lower extremity PNF pattern bilaterally using the D1 extension and flexion pattern, which was extension-abduction-internal rotation and flexion-adduction-external rotation. The patient was made to lie on the side of the treatment leg at the edge of the couch with the hip in flexion-adduction-external rotation and the knee flexed and the untreated knee extended. The therapist stood facing the patient and next to the extended leg while adjusting his position in response to the motion of the limb (Figure 2). The physiotherapist's proximal hand pushes down the anterior superior iliac spine of the untreated side to stabilize the pelvis. The therapist’s distal grip stretches, commands, resistance, and timing were the main areas of attention. Whereupon, the therapist instructed a 10-second isotonic contraction of the iliopsoas with emphasis on rotation. Over time, the resistance would increase. After the contraction has been held, the patient was instructed to relax. Gradually, both the patient and the therapist would loosen up. The limb would then be taken to the newly attained limit of the range and held for an additional 10 seconds, either passively or actively.

**Figure 3:**
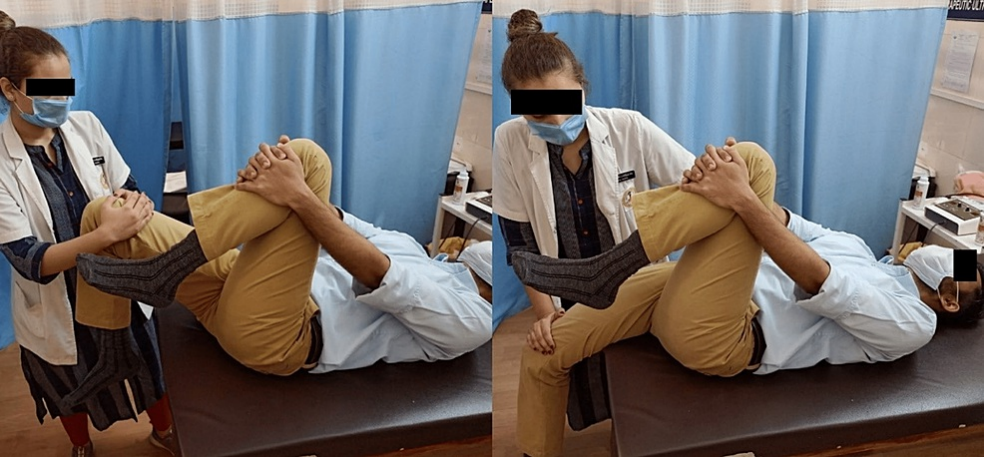
Start (left) and end (right) position for MET stretching technique MET: muscle energy technique

In group B, participants received basic conventional physiotherapy along with bilateral post-isometric relaxation stretching of the iliopsoas muscles three times in a single session with a break of two minutes in between each repetition. The patient was positioned in the supine test position with the buttocks at the edge of the couch, the opposing hip and knee fully extended, and the patient's hand holding them in place. The therapist positioned herself at the patient's foot end, facing the leg that would receive treatment, and leaving the other leg free to hang down (Figure 3). After 10 seconds of resistance, the therapist asked the patient to inhale and move the treatment leg toward her. After the isometric contraction, the leg was held for 30 seconds on the floor after being moved just slightly past the limit with a small amount of painless pressure. The right breathing techniques were demonstrated, which include inhaling during contraction, holding the breath during contraction, and exhaling once the contraction phase had ended and relaxation started.

## Results

The level of significance for the statistical analysis was set at p<0.05, and descriptive and inferential statistics were performed using the chi-square test, student paired and unpaired t-tests, and software from SPSS 27.0 (IBM SPSS, Armonk, NY) and GraphPad Prism 7.0 (GraphPad Software, San Diego, CA). The student t-test was used to compare groups A (PNF) and B (MET) in order to determine which treatment was most effective for lowering LBP, enhancing iliopsoas flexibility, and reducing lumbar lordosis angle. Within groups A and B, a paired t-test was used to compare pre and post-scores. An unpaired t-test was used to compare the post-mean difference scores between groups A and B. The subject characteristics for both groups are displayed in Table 1. The mean age (P<0.83) and sex (P<0.42) of the two groups were very marginally different.

**Table 1:** Participant characteristics

Baseline/subject characteristics	Group A	Group B	P-value
Age in years	37.72±11.84	37.20±12.39	0.83, NS
Gender: Male	19(38%)	24(48%)	0.41, NS
Gender: Female	31(62%)	26(52%)	

**Figure 4:**
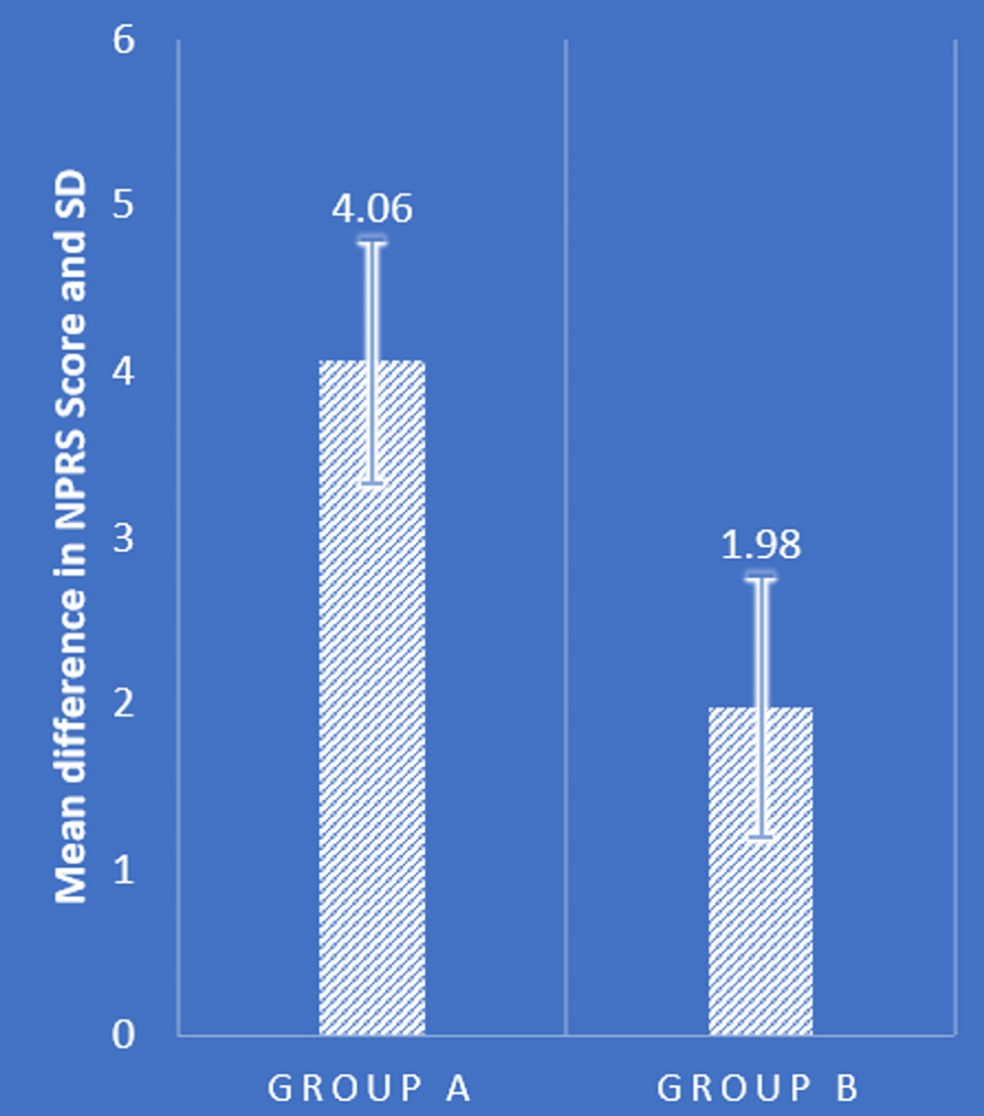
Comparison of mean difference in NPRS score in two groups NPRS: numerical pain rating scale; SD: standard deviation

**Figure 5:**
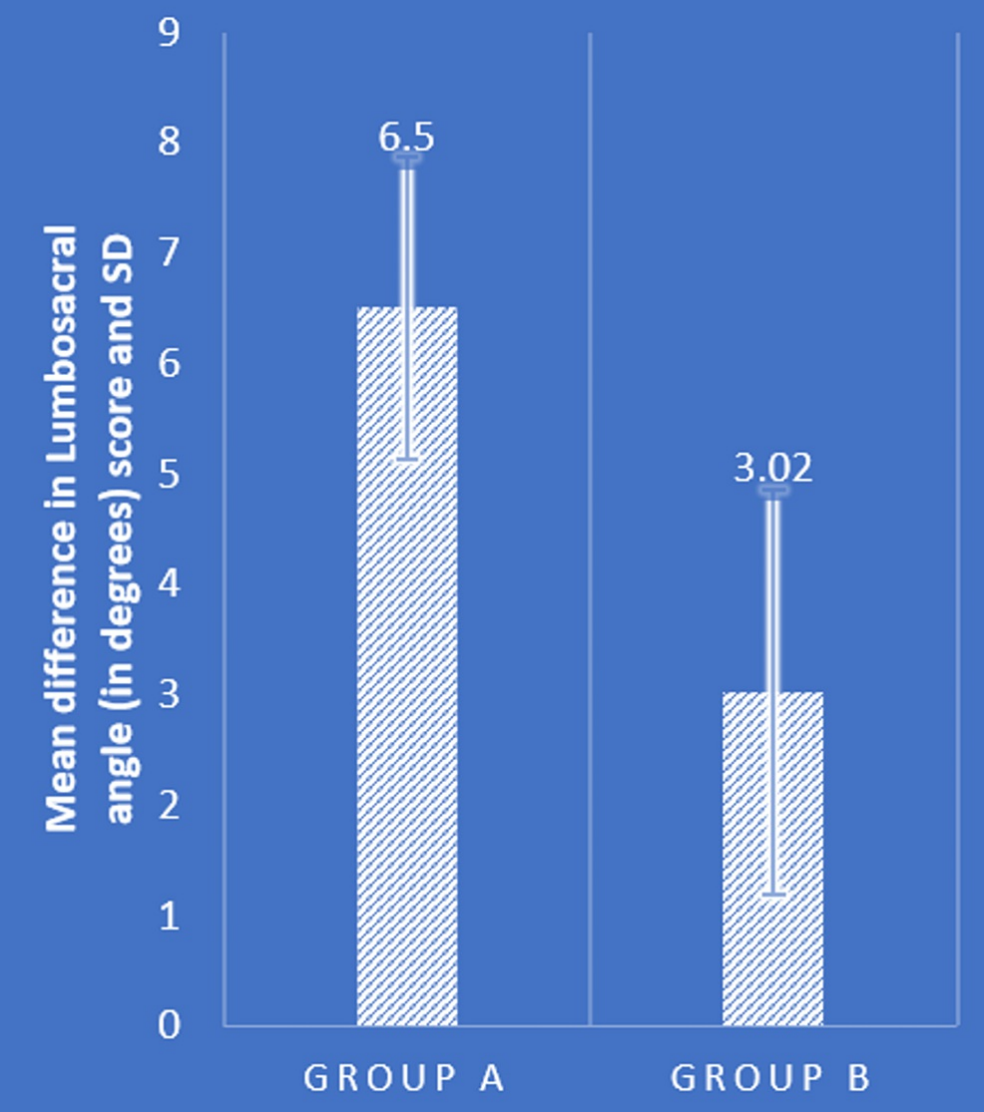
Comparison of mean difference in lumbosacral angle (in degrees) in two groups SD: standard deviation

**Figure 6:**
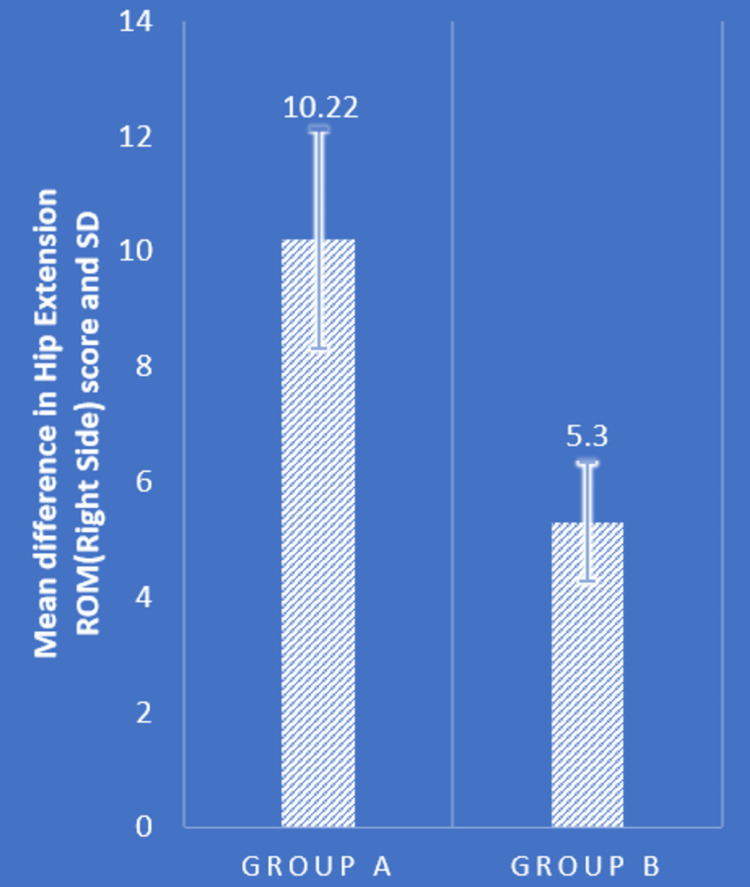
Comparison of mean difference in right hip extension ROM in two groups ROM: range of motion; SD: standard deviation

**Figure 7:**
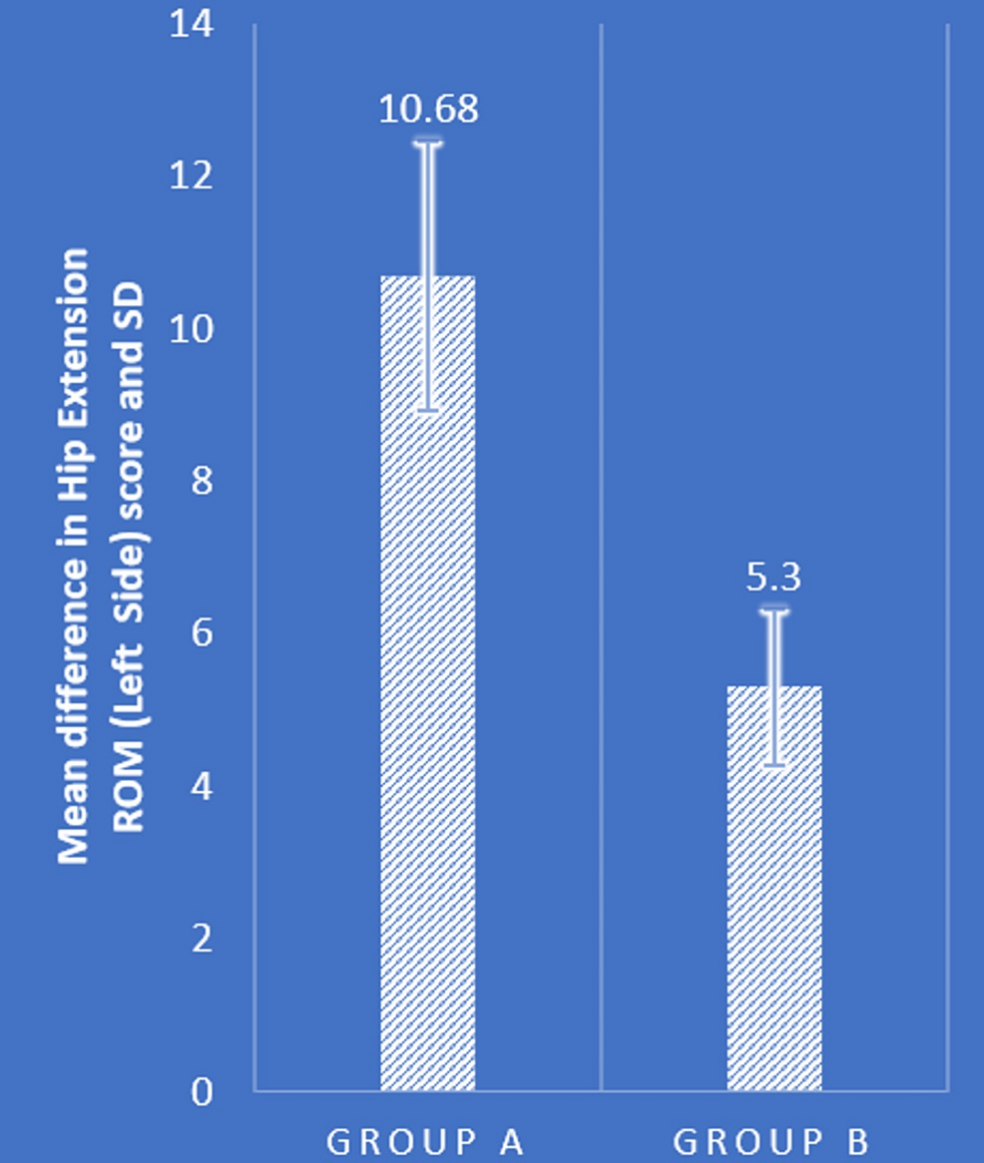
Comparison of mean difference in left hip extension ROM in two groups ROM: range of motion; SD: standard deviation

Table 2 and Figures 4-7 show statistical evidence for the impact of treatment on low back pain, the LSA, and the extension range of motion of the bilateral hip joints, respectively. Table 2 depicts a statistical analysis of the measured variables as well as the significant value of the comparison between the groups' pre- and post-intervention data. In groups A and B, there was a considerably lower level of pain, LSA, and right and left hip extension ROM following treatment compared to before treatment (P<0.0001). When the mean difference in LSA, hip extension ROM, and post-intervention pain range was compared between the two groups, the PNF technique performed better than the MET technique (P<0.0001), showing a significant difference between both but PNF > MET.

**Table 2:** Mean NPRS value, LSA angle and right and left hip extension range of motion pre and post treatment of groups A and B and between groups A and B NPRS: numerical pain rating scale, LSA: lumbosacral angle, ROM: range of motion, SD: standard deviation

Outcome measures	Group A		P-value	Group B		P-value	Mean difference (X±SD)		P-value
	Pre-treatment	Post-treatment		Pre-treatment	Post-treatment		Group A	Group B	
NPRS	6.20±1.39	2.14±1.19	0.0001	6.14±1.39	4.16±1.14	0.0001	4.06±0.73	1.98±0.79	0.0001
LSA (degrees)	46.86±2.95	40.36±2.03	0.0001	46.92±3.22	43.90±2.54	0.0001	6.50±1.37	3.02±1.83	0.0001
Right hip ROM (degrees)	14.84±2.53	25.06±3.61	0.0001	14.84±2.91	20.14±2.66	0.0001	10.22±1.90	5.30±1.03	0.0001
Left hip ROM (degrees)	10.56±2.50	21.24±2.49	0.0001	12.68±2.69	17.98±2.55	0.0001	10.68±1.77	5.30±1.03	0.0001

## Discussion

LBP is a notable factor in the decline of both the young and elderly population's quality of life. Since LBP is a chronic and serious condition, it must be treated as soon as possible with a thorough evaluation and treatment plan. Iliopsoas tightness causes lumbar hyper-lordosis and is one of the most common causes of LBP. According to Jun-Yong Lee's research, there was a significant difference in iliopsoas muscle tension between the low back pain patient group and the control group. He suggested that during physiotherapy sessions, more focus should be placed on releasing iliopsoas muscle tension [1]. In another study, the flexibility of the iliopsoas was improved in supine, knee semi-flexion, and lateral posture as a direct result of the stretching of this muscle. It also led to the retroversion and neutralization of the pelvis [12].

In this study, the effectiveness of PNF with MET stretching for the iliopsoas muscle in reducing low back pain in people with lumbar hyper-lordosis was tested. The difference between both the procedures is the position of the patient's lower limb, i.e., in PNF it is moved diagonally and in MET it is moved in line with the hip joint flexion axis; another difference is the isotonic contraction used in PNF and the isometric contraction used in MET. Both the PNF and MET groups showed a significant improvement in the study's outcome measures, but the PNF group's improvement was more pronounced. This is consistent with Marvin's observation that the PNF hold-relax group had significant improvements in range, which he believed could be explained by the active mobilization and autogenic inhibition of connective tissue [22]. In the studies on the immediate effects of hold-relax stretching of the iliopsoas, Malai et al. also found that it decreased lumbar lordosis and pain, improved transversus abdominis activation, and increased hip flexor length in patients with non-specific chronic LBP with lumbar hyper-lordosis [23].

By using voluntary muscle contraction and promoting muscle relaxation to prevent reflexive muscle contractions, PNF stretching techniques increase joint ROM. In order to increase flexibility, stretching techniques like hold-relax, contract-relax, slow reversal, etc., are now frequently used. They all call for the contraction and relaxation of agonist and antagonist muscles in succession. The ideal sequence is three repetitions of a 10-second active push phase followed by a 10-second passive rest phase. Theoretically, PNF stretching should be superior to static stretching methods because they stimulate not just muscle fibers but also sensory receptors in both the antagonist and agonist muscles [24].

The LSA, hip extension ROM, and pain were all improved in the MET group. METs are a set of typically painless mobilization techniques that are used to increase range of motion, decrease muscle spasm, decrease tissue edema, stretch fibrous tissue, and retrain the stabilizing function of inter-segmentally related muscles [25]. Although it has been tried in the past and found to be effective in treating musculoskeletal conditions in the low back and other parts of the body, there is not yet enough proof to recommend it for everyday use [26].

Three outcome measures, namely the NPRS, bilateral hip extension ROM, and LSA, were used to evaluate the effectiveness of both interventions. According to research, a 2-point shift on the NPRS used to measure low back pain indicates a clinically significant shift that exceeds the measurement error margin [17]. Lumbosacral angle measurement, which is regarded as the gold standard method for the purpose [19], was used to measure the lumbar lordosis angle. Bilateral hip extension ROM was measured using a validated goniometer for longitudinal exams in clinics in order to assess the tightness of the iliopsoas [21].

In this study, PNF and MET stretching were given thrice in a single session. All three outcome measures significantly improved for both groups. A comparison between the two groups revealed that the PNF group had significantly outperformed the MET group in terms of decreased lumbar lordosis angle, LBP, and increased hip extension range of motion (P 0.05), compared to MET, it had a quicker rate of influence, more people achieved iliopsoas muscle flexibility, and reduced lumbar lordosis angle. Because PNF involves better positioning and recruitment of the muscle that is intended to be stretched, it is more effective than MET.

The study has not included a follow-up time because the stretching technique is intended for the instant relief of low back pain, which occurs after sitting for a prolonged period due to iliopsoas tightness. The PNF stretching technique has outshone the MET technique in instantly relieving low back pain. The low back pain will eventually be back whenever the person sits for a prolonged period of time again and again stretching this muscle will relieve the pain.

The study could be expanded in the future to include an equal number of male and female patients, examine any other muscle in the body that occasionally experiences tightness, and run for a longer duration to determine the precise length of time required to treat tightness using either technique.

Limitations of the study were that only young, healthy people were included in it, a wide age range was taken into consideration for the study population, and unequal numbers of men and women were recruited.

## Conclusions

In today's lifestyle, especially sedentary ones, low back pain is a major concern. The causes of this back pain vary, but one of the most typical ones is increased lumbar lordosis in inactive people, which is brought on by iliopsoas tightness. The faster the tightness is released, the faster the LBP will resolve. We compared two widely used techniques in this study to determine which was the most effective. The hold-relax proprioceptive neuromuscular facilitation stretching technique and the post-isometric relaxation muscle energy stretching technique for the iliopsoas muscle were compared, and it was determined that both were effective in reducing lumbar lordosis and reducing pain in just one session. However, the latter's outcomes fell short of the former. To determine the precise time needed to treat tightness using either technique, the study could be expanded in the future to include an equal number of male and female patients, examine any other muscle in the body that occasionally experiences tightness, and run for a longer period of time.
